# Nanoscale zero-valent iron/persulfate enhanced upflow anaerobic sludge blanket reactor for dye removal: Insight into microbial metabolism and microbial community

**DOI:** 10.1038/srep44626

**Published:** 2017-03-16

**Authors:** Fei Pan, Xiaohan Zhong, Dongsheng Xia, Xianze Yin, Fan Li, Dongye Zhao, Haodong Ji, Wen Liu

**Affiliations:** 1 School of Environmental Engineering, Wuhan Textile University, Wuhan, 430073, P.R. China; 2 Engineering Research Centre for Clean Production of Textile Dyeing and Printing, Ministry of Education, Wuhan, 430073, P.R. China; 3 School of Materials Science and Engineering, Wuhan Textile University, Wuhan 430073, P.R. China; 4 Environmental Engineering Program, Department of Civil Engineering, Auburn University, Auburn, AL 36849, USA; 5 School of Civil and Environmental Engineering, Georgia Institute of Technology, Atlanta, GA 30332, USA

## Abstract

This study investigated the efficiency of nanoscale zero-valent iron combined with persulfate (NZVI/PS) for enhanced degradation of brilliant red X-3B in an upflow anaerobic sludge blanket (UASB) reactor, and examined the effects of NZVI/PS on anaerobic microbial communities during the treatment process. The addition of NZVI (0.5 g/L) greatly enhanced the decolourization rate of X-3B from 63.8% to 98.4%. The Biolog EcoPlate^TM^ technique was utilized to examine microbial metabolism in the reactor, and the Illumina MiSeq high-throughput sequencing revealed 22 phyla and 88 genera of the bacteria. The largest genera (*Lactococcus*) decreased from 33.03% to 7.94%, while the *Akkermansia* genera increased from 1.69% to 20.23% according to the abundance in the presence of 0.2 g/L NZVI during the biological treatment process. Meanwhile, three strains were isolated from the sludge in the UASB reactors and identified by 16 S rRNA analysis. The distribution of three strains was consistent with the results from the Illumina MiSeq high throughput sequencing. The X-ray photoelectron spectroscopy results indicated that Fe(0) was transformed into Fe(II)/Fe(III) during the treatment process, which are beneficial for the microorganism growth, and thus promoting their metabolic processes and microbial community.

As the world’s population continues to grow, more and more wastewater is produced^1^, and accordingly, developing more effective wastewater treatment technologies has become one of the major challenges of modern society^2^. Conventional wastewater treatment plants (WWTPs) technology such as upflow anaerobic sludge blanket (UASB) has been demonstrated effective in treating some industrial wastewater (e.g. medicine and brewery wastewater) in the past decades^3^,^4^.

Oxidation of contaminants using persulfate (PS) is one of the emerging advanced oxidation processes (AOPs), which has attracted increasing interest in the wastewater treatment field^5^. Typically, PS can serve as a source of sulfate radicals (SO_4_^−^•) due to its high solubility, good stability, high redox potential (2.01 V) and low cost^6^. Sulfate radicals (SO_4_^−^•) can highly effectively degrade various organic contaminants (dyes, phenolics, and antibiotics) in water^7^. Typically, SO_4_^−^• radicals are formed through activation of PS by transition metal catalysts (e.g. Fe^2+^)^5^. In addition, zero-valent iron (ZVI) particles as the source of Fe^2+^ have been used for activating PS under both anaerobic and aerobic conditions^8^,^9^. Moreover, iron was one of the most prominent additive substances to improve anaerobic digestion performance considering its low price and conductive properties^10^,^11^,^12^. Zhang *et al*.^13^ found that ZVI could promote the performance of UASB reactor on degradation of azo dye at low hydraulic retention time (12 h) and low temperature (25 °C). Some reports also proved that nanoscale zero-valent iron (NZVI) can accelerate the fermentation and hydrolysis stages for its action as electron donor^14^,^15^.

Nanoparticles (NPs) can interact with biological systems and humans, when released into the environment^16^. Many studies have reported that NPs could accumulate in aquatic organisms and cells, which would lead to stress or even mortality^17^,^18^,^19^. Metal nanoparticles, a major type of NPs, have shown strong antimicrobial effects for microorganisms^20^. For example, the antimicrobial activity of iron-based nanoparticles against bacteriophage^21^ and *Escherichia coli (E. coli*)^22^,^23^ has been reported. NZVI could cause serious damage to the respiratory activity and to the integrity of the cell membrane of *E. coli* in the absence of oxygen^22^,^23^,^24^. Therefore, more attention should be paid to the application and disposal of manufactured NPs, so as to avoid unintended environmental harms. Although previous works studied removal of contaminants in the ZVI/PS system, little information is known about the effects of NZVI/PS on bio-communities and organisms, and the underlying mechanism also should be figured out. Moreover, there is limited information on the environmental fate of most concerned NPs (e.g. NZVI) in WWTPs (e.g. UASB or SBR reactor).

Biolog EcoPlates^TM^ has been used to analyze the bacterial communities in various environments^25^,^26^, and the Illumina MiSeq sequencing technique has been widely used for analyzing the microbial functional and species in many environments samples^27^. By means of these molecular biological methods, we can better understand the bacterial communities in various environments.

In this study, we evaluated the efficiency of NZVI/PS enhanced degradation of brilliant red X-3B in a UASB reactor, and determined the effects of NZVI/PS on anaerobic microbial communities in the reactor system. The main objectives were to: (1) test degradation efficiency of brilliant red X-3B by NZVI activated PS, (2) examine the performance of UASB combined with NZVI/PS for degradation of brilliant red X-3B and the chemical oxygen demand (COD) removal, (3) determine the temporal and spatial changes in microbial metabolism of anaerobic microorganisms in UASB through Biolog EcoPlate^TM^ test^28^ following the addition of NZVI/PS; and (4) analyse the change of microbial communities of the anaerobic microorganisms in UASB using the Illumina MiSeq sequencing technique in the presence of NZVI/PS. The results may facilitate improved degradation efficacy of persistent organic substances such as dyestuffs by incorporating nanotechnologies into biological treatment processes in WWTPs.

## Results and Discussion

### Degradation of reactive brilliant red X-3B by NZVI/PS enhanced UASB reactor

**Figure 1:**
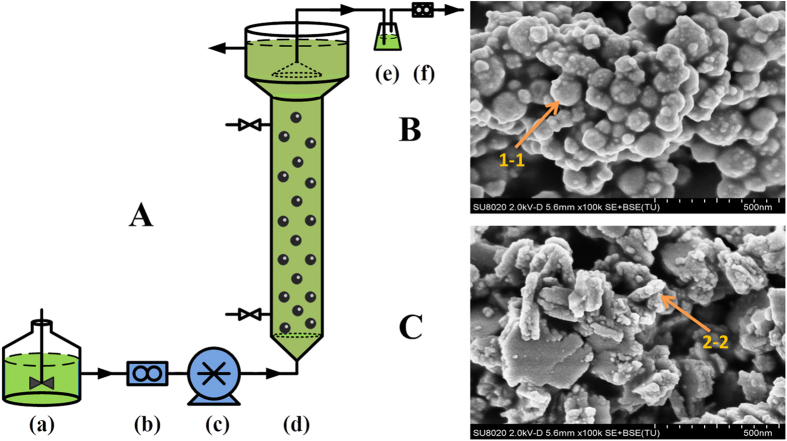
(**A)** Schematic diagram of the UASB reactor setup. (a) feed tank, (b) flow counter, (c) peristaltic pump, (d) reactor, (e) water-sealed bottle, and (f) wet gas flow meter. SEM images of **(B)** fresh and **(C)** spent NZVI in UASB reactors.

Figure 1A shows the schematic diagram of the UASB reactor, and Fig. 1B,C show the SEM images of NZVI. Fresh NZVI appeared as aggregated nanospheres with individual sizes of 50–100 nm (Fig. 1B). After reaction in the UASB reactor for 30 days (Fig. 1C), the nanospheres were transformed into larger bulk aggregates, with some rod-like microorganisms attached on the materials. The EDS line-scanning indicated that Fe was the main element for fresh NZVI (through sections 1–1′) (Figure S1A), while O accounted for a large percentage for used NZVI (Figure S1B) (through sections 2–2′), suggesting oxidation of Fe(0) during the reaction. In addition, the presence of C indicated the attachment of microorganisms.

**Figure 2:**
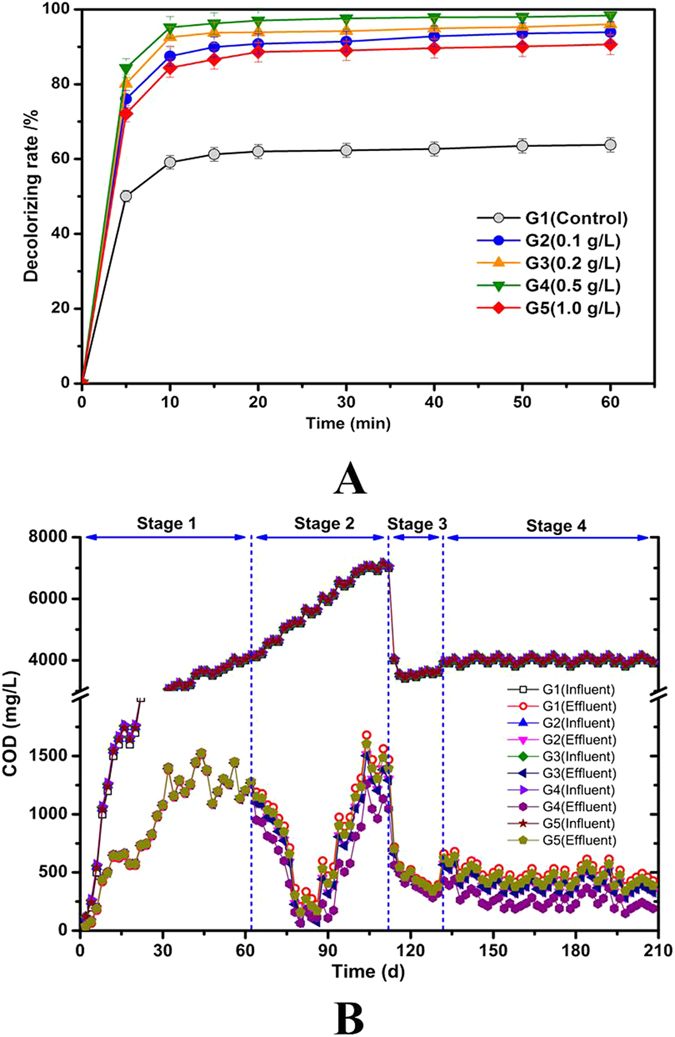
(**A**) Decolorization rates in UASB reactors with different NZVI dosages. **(B)** COD removal in UASB reactors with different NZVI dosages.

Table 1 gives the COD removal efficiency, decolorization ratio and COD in influent and effluent concentration in stable operation stage of UASB reactor. The addition of NZVI into the sludge greatly affected the UASB reactor process. Low dosages (0.1 g/L, 0.2 g/L and 0.5 g/L) of NZVI remarkably promoted the removal of COD and X-3B. However, an excessive dosage (1.0 g/L) of NZVI would pose a negative impact on the UASB reactor performance. Figure 2A shows the decolourization rates in the UASB systems with different NZVI/PS dosages. At an NZVI dosage of 0.1 g/L, the decolorization extent was increased from 63.8% to 93.9% at 60 min. Further increasing the NZVI dosage to 0.5 g/L enhanced the decolorization rate to 98.4%, and the decolourization was decreased to 90.6% at a higher dose of 1.0 g/L NZVI. As shown in Fig. 2B, the COD removal was presented distinct effects after addition of NZVI. When compared with reactor G1 which was operated without NZVI, all of reactors (G2-G5) showed better COD removal (88.57% − 93.53%) during the stage of stable operation. Moreover, Fig. 2B showed that the maximum COD removal occurred at a concentration of 0.5 g/L and that the minimum COD removal occurred at a concentration of 1.0 g/L with NZVI range (from 0.1 g/L to 1.0 g/L).

Le *et al*.^29^ found that 100% color removal efficiency and 54% TOC removal efficiency were achieved in 45 min with an initial dye Reactive Blue 19 concentration of 0.1 mM under typical conditions (pH 7.0, 0.8 g•L^−1^ ZVI, 10 mM persulfate and 30 °C). Rodriguez *et al*.^30^ found that complete azo dye Orange G (OG) removal was achieved within the first 30 min when PS was activated by Fe(II), but the longer reaction time required when ZVI was employed. In this study, NZVI and PS were firstly added into the feed tanks of the reactors full of dyeing wastewater and the following stoichiometric reactions between NZVI and PS were initiated immediately in Equations (1, 2, 3, 4, 5)^31^,^32^,^33^,^34^,^35^.


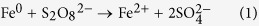



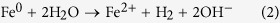



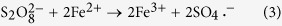



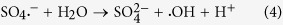



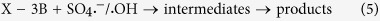


The formed sulfate radicals (SO_4_•^−^) and hydroxyl radicals (•OH) are strong reactive oxygen species (ROS), which can efficiently degrade X-3B. The dyeing wastewater with NZVI and PS in the five feed tanks was continuously agitated by a constant stirrer with a 200 rpm of mixing for 60 min, while the degradation of organic pollutants using NZVI/PS may take only a few minutes^14^. Moreover, it was reported that the formed sulfate radicals (SO_4_•^−^) could react with S_2_O_8_^2−^ to form SO_4_^2−^ and S_2_O_8_•^−^ under acidic conditions^36^, and SO_4_•^−^ only existed in aqueous-phase atmosphere for instance in cloud droplets^37^. In addition, iron could be initiated in anoxic environments^38^. Therefore, the reaction of PS and NZVI occurred in the feed tank, while the biological degradation process on X-3B and the reaction of NZVI with sludge mainly occurred in UASB reactors. It is a self-consumption reaction for ZVI with regarding to the reactions 1–5, and transformation of Fe(0) was later confirmed by XPS in the later Section. Therefore, reuse of reacted material for continuous cycle of reaction should not be available, while re-addition of ZVI and PS after initialization of UASB should be a better choice in real application for economic consideration. Fe-based materials including NZVI can also enhance the biological process in UASB due to the release of Fe^2+^/Fe^3+^, which are essential elements and beneficial for the microbial growth^39^. Furthermore, NZVI may affect the microbial metabolism and communities, and that the microbial degradation effectiveness (to be discussed in detail in the following sections). However, high dosages of NZVI may cause a potential toxicity to bacteria^40^, the addition of NZVI in anammox UASB reactors favored the growth of anammox bacteria and promoted their aggregation in flocs^41^.

### Microbial metabolism

**Figure 3:**
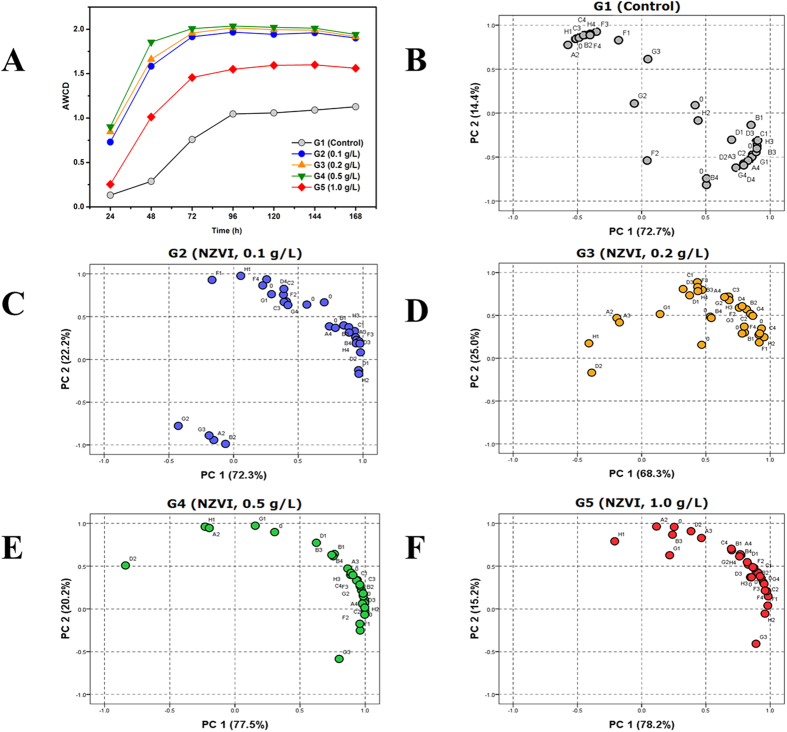
Ordination diagrams of CLPPs from principal component analysis of carbon source utilization profiles in five UASB reactors.

The community-level physiological profiling (CLPP) of microbial communities could be represented by the average well color development (AWCD) of Biolog EcoPlate^TM^ technique^25^,^42^. Figure 3A shows the AWCD changes of the 5 groups of UASB reactors. The results indicate that the CLPP, which reflects the substrate utilization patterns by microbial communities, varied significantly in the UASB reactors in response to the NZVI additions. In contrast, the AWCD curves of 5 UASB reactors showed similar patterns. The microbial activities of G2, G3 and G4 were higher than those of G1and G5 in the initial 24 h, with G1 (the control) showing the lowest microbial activity. In all cases, the microbial activity increased sharply at 96 h, and then reached a moderately stable stage after 120 h. Among the 5 UASB reactors, the much greater microbial activities of G2-G5 over G1 (Control) clearly indicates that NZVI could greatly improve CLPP, which also explains the improved COD removal by the addition of NZVI in the UASB reactors (Table 1). Among the UASB reactors which received the different doses of NZVI, the CLPP of G2-G4 increased slightly with increasing NZVI dose, while that of G5 was lowered. Figure 3A shows that the maximum CLPP occurred at 0.5 g/L of NZVI while the minimum CLPP occurred at 1.0 g/L in the different NZVI ranges (from 0.1 g/L to 1.0 g/L). Although addition of NZVI with all dosages promoted the CLPP, the CLPP was firstly increased from 0.1 g/L to 0.5 g/L, while then decreased with NZVI further increasing from 0.5 g/L to 1.0 g/L. The optical density (OD) data, which were obtained from Biolog EcoPlate^TM^ assay, were normalized and subjected to PCA to study the impact of NZVI on the CLPP of microbial communities^25^. Figure 3B–F show the ordination diagrams of CLPPs from the principal component analysis (PC1 and PC2) of 31 carbon source utilization profiles in five UASB reactors, which reflects the change in the utilization pattern of carbon sources substrates of the microbial communities in response to after the different NZVI doses. It is evident from the PC1-PC2 diagrams that the carbon source substrates are mostly found in the lower right and upper left regions for the control reactor G1 (without NZVI), while the are mainly located in the lower left and the upper right areas for G2-G5. These observations indicate that the addition of NZVI notably affects the utilization patterns of carbon source substrates.

In general, a low utilization of carbon source substrates has a negative loading on PC1, while a high utilization of carbon source substrates would present a high loading on PC1 (Fig. 3B–F).The variability on PC1 can be explained by the change in the substrate utilization patterns by the microbial communities, such as carboxylic acids, carbohydrates, phenolic compounds, polymers and amino acids^25^. The microbial communities in G1 favored utilization of carbohydrates (C2, D2, and E2), and amino acids (A4, D4, and F4). in contrast, the addition of NZVI (0.1 g/L, 0.2 g/L and 0.5 g/L) showed relatively high utilization of carboxylic acids (A3, B3, D3, E3, F3, F2, and H3), polymers (C1, D1, and E1) and amines/amides(G4 and H4), indicating that NZVI could promote the metabolism of the microbial communities. The favorable impact of NZVI on the microbial communities in the UASB reactors could be due to improved sulfate reduction and methane production^43^. Indeed, it was reported that the microbial communities, particularly homoacetogenic bacteria and propionate-utilizing bacteria, could be enhanced with the addition of ZVI^44^. Moreover, in the presence of low concentrations of NZVI, the microbial communities are able to mitigates to toxic effects and overcome the oxidative stress associated with the ROS through mechanisms such as the production of extracellular or intracellular substances^45^, and/or some kind of cell repair mechanisms^25^. However, further investigations are needed to confirm these assertions.

In contrast, at 1.0 g/L NZVI, the utilizations of amino acids (B4, C4, and F4), amines/amides (G4 and H4), carboxylic acids (C3 and H3), and carbohydrates (B2 and C2) were all decreased. This observation explains the inhibitive effect of NZVI at elevated doses on the microbial communities. The results agree with the reported data that 1.0 g/L NZVI may pose toxic effects on indigenous bacteria in bioremediation processes^46^,^47^.

### MiSeq-pyrosequencing results and microbial community structures

Figure 2A shows that the decolorization ratio was significantly changed upon the addition of different doses of NZVI. The decolorization ratios of G2 and G5 were similar, and those of G3 and G4 were comparable. According to the decolorization ratios and the NZVI dosages, samples collected G1, G3 and G5 were subjected to the Illumina pyrosequencing.

The total of 223288 effective 16 S rRNA sequence reads (51836, 101098, and 70354 for G1, G3 and G5, respectively) were generated by the Illumina MiSeq high-throughput sequencing of the samples from the corresponding UASB reactors. Table 2 lists the sequence information on the 3 samples. The Simpson/Shannon diversity level and Chao1/Ace estimator are used to reflect the microbial communities’ phylotype richness levels. Table 2 shows that the Chao1/Ace values at low NZVI doses (0.2 g/L) (G3) and high NZVI doses (1.0 g/L) (G5) were both higher than that for the control (G1). Rarefaction analyses were then performed to compare and standardize the detected taxon richness among the samples^48^. Figure S2 in the Supporting Information (SI) presents the rarefaction curves. The rank abundance curves in Figure S3 in SI show that G1, G3 and G5 all had extremely abundant microbes, though G3 and G5 exceeded G1 in both microbial diversity and richness. As indicated in the Venn diagram in Figure S4 in SI, 1,690 OTU species occupied group G1, 2,678 OUT species occupied group G3, and 1,982 OTU species occupied group G5. However, the Venn diagram indicated that only 7.83% (497 OTUs) OUT species of the total OTUs were common for the three samples, and the shared OTUs between G1 and G3, G1and G5, and G3 and G5 were 11.07%, 10.27% and 13.07%, respectively, indicating that the microbial compositions among G1, G3 and G5 were quite different.

### Taxonomic complexities of bacterial communities

**Figure 4:**
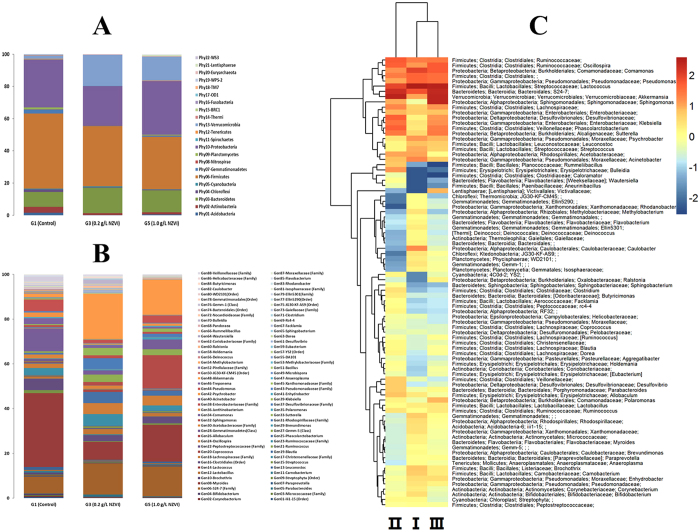
(**A**) Distribution of bacterial community structure at phylum levels (abundance ≥ 0.1%) in G1, G3 and G5 UASB reactors; **(B)** Distribution of bacterial community structure at genus levels (abundance ≥ 0.1%) in G1,G3 and G5 UASB reactors. **(C)** Microbial community Heat-map of the classified genera (abundance ≥ 0.2%) in G1, G3 and G5 UASB reactors.

The functions and behaviors of bacteria in microbial communities influence the biomass and microbial activity^49^, The bacteria from G1, G3 and G5 displayed greatly different abundance levels. Figure 4A illustrates the bacterial community abundance levels of G1, G3 and G5 at the phylum level. A total of 22 phyla were identified in the bacteria from the 3 samples. The classified phyla could be separated into five branches based on the species distribution in the 3 samples: Phy01-Phy14 (present in all 3 samples), Phy15-Phy19 (only in G1), and Phy20 (in G3 and G5), Phy21 (only in G5) and Phy22 (in G1and G5).

Phy03-*Bacteroidetes*, Phy06-*Firmicutes*, Phy10-*Proteobacteria* and Phy13-*Verrucomicrobia* were the dominant phyla in the bacteria communities from the 3 samples, and the 4 dominant phyla differed distinctively in the 3 samples. The percentages of these phyla in G1, G3 and G5 were measured at: Phy03-*Bacteroidetes* = 9.3%, 15.8% and 13.7%, Phy06-*Firmicutes* 46.7%, 37.3% and 32.5%, Phy10-*Proteobacteria* = 29.6%, 24.4% and 33.3%, and Phy13-*Verrucomicrobia* = 1.9%, 19.2% and 14.9%, respectively. Thus, Phy06-*Firmicutes* were weakened while Phy03-*Bacteroidetes* and Phy13-*Verrucomicrobia* were enriched with the NZVI treatments. However, Phy10-*Proteobacteria* was weakened at the low dose of NZVI (G3) while enriched at the high dose of NZVI (G5). The *Bacteroidetes* phylum is a large group of bacteria, which are found in many aquatic systems, revealing a high level of metabolic and phenotypic diversity^50^. The enrichment of *Bacteroidetes* phylum is likely to play a role in improving the anaerobic degradation of the dye stuff in the UASB reactors.

The microbial community’s abundance was further investigated at the genera level (Fig. 4B). A total of 88 genera were identified in the bacteria from the 3 samples. The classified genera are separated into six branches based on the species distribution in the 3 samples: Gen01-Gen48 (existed in all 3 samples), Gen49-Gen56 (only in G1), Gen57-Gen63 (in G3 and G5), Gen64-Gen71 (only in G3), Gen72-Gen83 (in G1 and G5) and Gen84-Gen88 (in G1 and G3). Gen05-*S24-7* (Family), Gen14-*Lactococcus*, Gen32-*Sphingomonas*, Gen34-*Comamonas* and Gen48-*Akkermansia* were the dominant genera in the bacteria from the 3 samples, in which the dominant genera differed markedly.

The percentages of these genera in G1, G3 and G5 were: Gen14-*Lactococcus* = 33.03%, 7.94% and 16.49%, Gen48-*Akkermansia* = 1.69%, 20.23% and 15.74%, Gen34-*Comamonas* = 10.15%, 1.91% and 4.39%, Gen32-*Sphingomonas* = 0.11%, 1.48% and 14.88%, *S24-7* (Family) = 7.55%, 13.88% and 13.38%, respectively. Evidently, Gen14-*Lactococcus* and Gen34-*Comamonas* were weakened with the NZVI treatments, while Gen48-*Akkermansia*, Gen05-*S24-7* (Family) and Gen32-*Sphingomonas* were enriched. *Akkermansia* are well known to utilize mucins (sulfate groups and complex glycoproteins) as nitrogen, sole carbon and energy sources^51^,^52^, and were found to exist in greater abundance with higher metagenome richness^53^. The increase in *Akkermansia* abundance by NZVI benefited to the metabolic profile of the microbial community^54^. The increase in *S24-7* (Family) abundance favored the metabolism of microbial community. *Sphingomonas* was found having great exuberant vitality and intense ability to fit for the environment^55^.

This bacterial community was further examined with the heat-map to recognize differences and similarities among the 3 samples. As presented in Fig. 4c, 86 bacterial genera in the 3 samples were recognized at the 0.2% abundance level, indicating the remarkably different microbial community structures in G1, G3 and G5.

The genera of the 3 samples in Fig. 4C were divided into four branches based on the species richness. From the top to the bottom of Fig. 4c, the first branch covers *Ruminococcaceae* to *Acinetobacter* with 20 genera, the second covers *Rummeliibacillus* to *Peptostreptococcaceae* with 66 genera, the third covers *Ruminococcaceae* to *Aneurinibacillus* with 25 genera, and the fourth covers *Victivallaceae* to *Peptostreptococcaceae* with 61 genera. According to Fig. 4c, the first branch was a high abundance branch in G1 and G5, while the second branch was a low abundance branch in G1 and G5, the third branch was a high abundance branch in G3, and the fourth branch was a low abundance branch in G3.

### Functional microbial strain analysis

**Figure 5:**
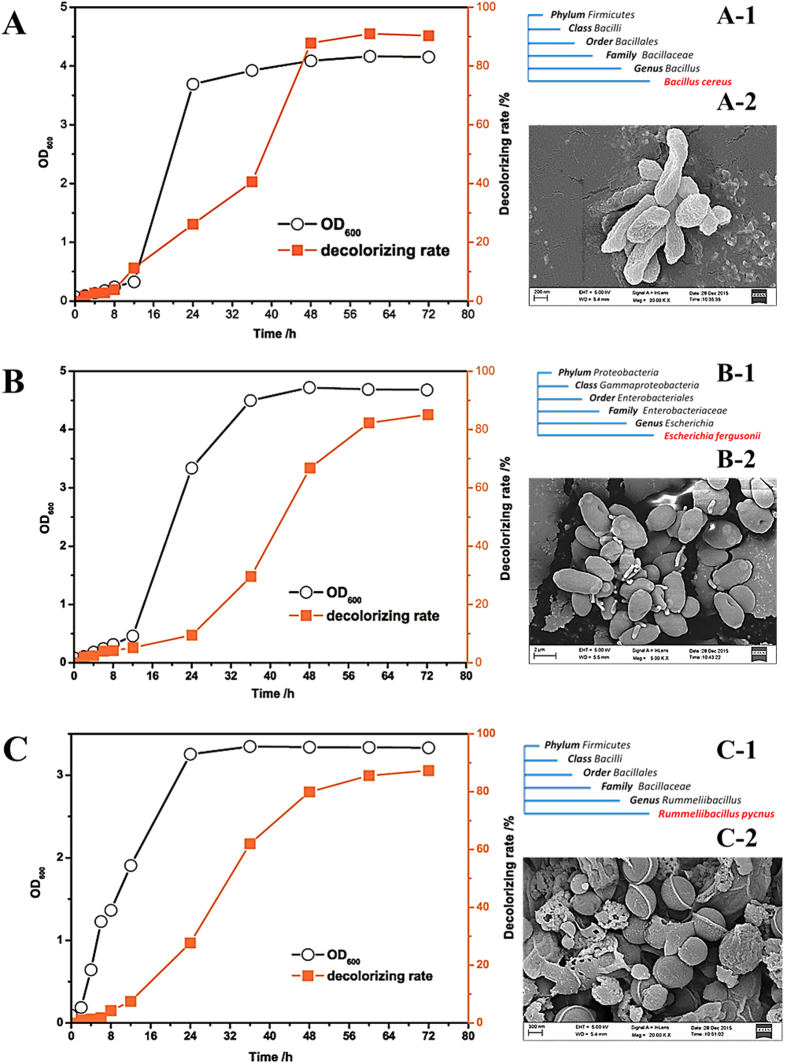
(**A**) the growth curve and biological degradation curve of, **(A-1)** the taxon of three strains and **(A-2)** the morphology of KX421197 (FP-A1); (**B**) the growth curve and biological degradation curve of, **(B-1)** the taxon of the three strains and (**B-2**) the morphology of KX421198 (FP-B1); (**C**) the growth curve and biological degradation curve of, (**C-1**) the taxon of three and **(C-2)** the morphology of KX421199 (FP-C1).

Two strains were isolated from G1, one strain was isolated from G2, two strains were isolated from G3, one strain was isolated from G4 and one strain was isolated from G5. Some of the seven strains were identified by 16S rRNA as the same strain and three different strains were finally identified. Then the three different strains were denoted as FP-A1, FP-B1 and FP-C1. FP-A1 was only present in G1; FP-B1 was present in all of 5 samples, whereas FP-C1 was only present in G3. All the three strains are able to use reactive brilliant red X-3B as their sole carbon and energy source, thereby bio-catalyzing the degradation of dye X-3B. Figure 5A–C shows the growth rates and the biological degradation rates for the three strains.

The three strains were further identified as *Bacillus cereus* (FP-A1), *Escherichia fergusonii* (FP-B1) and *Rummeliibacillus pycnus* (FP-C1) by 16S rRNA analysis and the taxon of three strains were shown in Fig. 5A-1–C-1. The NCBI Numbers of three strains were KX421197 (FP-A1), KX421198 (FP-B1) and KX421199 (FP-C1); the phylogenetic trees of three strains are presented in Figures S5–S7 in SI, and the morphologies of the three strains, which were observed under FE-SEM, are given in Fig. 5A-2–C-2. Moreover, the results from high-throughput sequencing also showed the genera of three strains as dye-degrading bacterial genera. *Bacillus cereus* belongs to *Bacillus* (genus) and it was found only in G1 (Fig. 4B); *Escherichia fergusonii* belongs to *Enterobacteriaceae* (Family) and it existed in all the three samples (Fig. 4B); *Rummeliibacillus pycnus* belongs to *Rummeliibacillus* (genus) and it was observed in G3 only (Fig. 4B). The distribution of three strains was consistent with that of genera in Fig. 4B. As a result, the high-throughput sequencing results were consistent with those from the bacterial strain analysis.

Nowadays, the microbiome studies by high-throughput sequencing of 16S rRNA gene, e.g., Polony sequencing technology, 454 pyrosequencing technology and Illumina pyrosequencing technology, have attracted great interests^56^. However, the reported results are usually difficult to reproduce across investigations which are caused by the large variation in the complex multistep process of 16S rRNA-based microbiome analysis, such as sample collection, DNA extraction, and purification, Polymerase chain reaction (PCR) process, sequencing and bioinformatics. In this study, we presented the conventional microbial isolation and pure culture, which should be a useful tool to confirm the results of high-throughput 16S rRNA gene sequencing.

### Mechanisms for effects of NZVI/PS on UASB efficiency

Based on the above results, the addition of NZVI/PS can affect the performance of UASB reactors, leading to much enhanced X-3B decolourization rate (Fig. 2A). Following the addition of NZVI/PS, remarkable changes in the microbial community in the UASB reactors were observed (Figs 3 and 4), i.e. a higher CLPP of microbial communities (Fig. 3) and much enhanced microbial communities, particularly homoacetogenic bacteria, and propionate-utilizing bacteria^44^. Moreover, the predominant microorganisms in the UASB reactors were changed. Specifically, (1) the competing bacteria (e.g., Phy06-*Firmicutes*) were inhibited, leading to enhanced microbial diversity; (2) Phy03-*Bacteroidetes* and Phy13- *Verrucomicrobia* in the UASB reactors were enriched, leading to a higher level of metabolic and phenotypic diversity^50^; and (3) the increases of Gen48- *Akkermansia*, Gen05-*S24-7* (Family) and Gen32-*Sphingomonas* bacteria with the addition of NZVI in UASB reactor are also beneficial to the biological degradation process.

**Figure 6:**
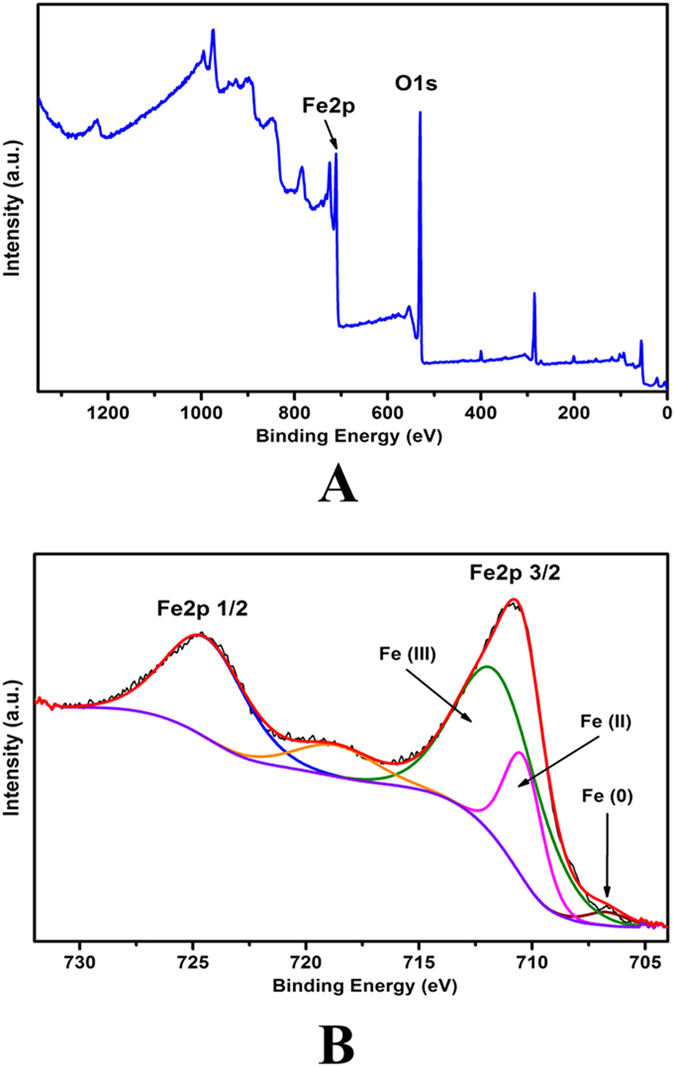
XPS spectra of reacted NZVI collected from UASB reactors: **(A)** Survey and **(B)** high resolution of Fe 2p.

By comparing with the control without the addition of NZVI, it is evident that the change in microbial community is attributed to the NZVI effects and may result from the modulation of NZVI. Figure 6 shows XPS spectra of spent NZVI after 30 days’ reactions in the UASB reactors. The main elements of used NZVI included: Fe (55.4%), O (35.2%) and C (9.4%). The much elevated O percentage indicates oxidation of Fe(0) during the reaction, while the presence of C on the materials manifests interactions of the microorganisms with NZVI. The two major peaks at 724.4 ± 0.2 eV and 710.8 ± 0.2 eV are assigned to Fe 2p_1/2_ and Fe 2p_3/2_, respectively. The three peaks at 706.5, 710.5 and 712.4 eV with a peak area ratio of 2.9:24.1:73.0 are attributed to Fe(0), Fe(II) and Fe(III) oxidation states^57^,^58^, respectively (Fig. 6B). Therefore, most of Fe(0) was transformed into Fe(II) and Fe(III), which are beneficial species for the microorganism growth. Therefore, the production of Fe(II)/Fe(III) and the associated effects on the biological growth and microbial community can be also responsible for the enhanced biodegradation of the dyestuff. In addition, the formed Fe(II) plays an important role for the formation of sulfate radicals (Equation (3)), thus promoting the degradation of X-3B. Moreover, the dissolve irons (Fe(II) and Fe(III)) may also catalyze the decomposition rate of X-3B. The roles of different iron ions should be further investigated in the future studies.

## Material and Methods

### Experimental methods

Five UASB reactors (G1-G5) were employed. PS was added into the UASB reactors (G1-G5) at 0.125 g/L. The G1 reactor was used as the control without addition of NZVI, and the G2-G5 reactors were subjected to various NZVI dosages (0.1, 0.2. 0.5 and 1.0 g/L). Figure 1A shows the schematic diagram of the UASB reactors and the detailed information of the reactors is provided in Text S1 in SI.

All reagents were obtained in high-purity and were used as received, including NZVI (≥99.9%, Aladdin Industrial Inc. Shanghai, China), Potassium persulfate (≥99.0%, Aladdin Industrial Inc. Shanghai, China), Reactive Brilliant Red X-3B (Sinapharm Chemical reagent Co., Ltd. Shanghai, China).

### Inoculated sludge and synthetic dyeing wastewater

Inoculated sludge was taken from anaerobic digesters of the sewage treatment plant based at Wuhan Textile University (Wuhan, China). The floc sizes of the inoculated sludge ranged from ~1.0 to ~3.0 mm (Figure S8, SI) and pH from 6.5 to 7.2. The detailed information of the inoculated sludge and the influent dyeing wastewater are provided in Text S2 in SI.

### Degradation of reactive brilliant red X-3B by NZVI/PS enhanced UASB system

In this study, the dyeing wastewater was firstly purged with high-purity nitrogen gas at a constant flow rate of 0.5 L/min for 5 min to remove oxygen and then was pumped into UASB reactor. The UASB reactors were first operated with a influent COD from 100 to 4,000 g/(m^3^•d) for two months for the pre-acclimation of the anaerobic microbial communities. COD levels in both influent and effluent were measured according to the standard method. Afterward, the UASB reactors were operated over four stages, i.e., start-up (0-62 d), X-3B loading increase (63-112 d), recovery (113-131 d) and stable operation (132-208 d). High-purity nitrogen gas was purged into the UASB reactor at a constant flow rate of 0.5 L/min for 20 min to maintain the DO concentration every 24 h during the reaction processes. And the DO concentration in UASB reactor was ranged from 0.18 to 0.20 mg/L. On Day 63, PS was firstly added into the five feed tanks of the reactors (G1-G5) which were full of dyeing wastewaters with the same concentration of 0.125 g/L. Afterwards, NZVI was added at dosage of 0.1, 0.2, 0.5 and 1.0 g/L into the four tanks of the G2-G5 reactors. During the reaction, the dyeing wastewater with NZVI and PS in the five feed tanks were continuously agitated by a constant stirrer with a 200 rpm of mixing for 60 min. Consequently, the dyeing wastewaters were pumped into UASB reactors by a peristaltic pump after purging with high-purity nitrogen gas at a constant flow rate of 0.5 L/min for 5 min to remove oxygen. On the other hand, the remaining NZVI was firstly took out from the feed tank and then putted into the reaction zone of UASB reactor, which was then fixed with anaerobic sludge.

Table S1 in SI provides the experimental schedule for influent COD and X-3B loading. The pH of dyeing wastewater in the UASB reactors was adjusted by 1.0 mol/L NaOH and 1.0 mol/L H_2_SO_4_, and pH was kept from 6.5 to 7.2. The operation temperature of the UASB reactors was maintained at 30 ± 2 °C, the hydraulic retention time (HRT) was kept at 18 ± 2 h, and the average cell residence time ranged from 20 to 40 d. On Day 198, samples were collected from each UASB reactors for further analyses.

The morphology and elemental percentages of NZVI in the UASB reactors was obtained on an SU8020 Ultra-High-Resolution field emission scanning electron microscope (FE-SEM) (Hitachi, Tokyo, Japan), and energy dispersive spectra were collected at the same time. X-ray photoelectron spectroscopy (XPS) of NZVI was conducted using a Thermo ESCALAB 250XI Multifunctional imaging electron spectrometer (Thermo Fisher Scientific, Waltham, USA). The microbial community-level physiological profiling was performed through the Biolog EcoPlate^TM^ test^26^. The anaerobic microorganism genera were identified via Illumina MiSeq high-throughput sequencing.

### Microbial community-level physiological profile (CLPP) determination

The Biolog EcoPlate^TM^ technique was employed to evaluate the microbial community physiological metabolic characteristics by assessing utilization of separate sole carbon source substrates distributed in a 96-well plate during 7 days incubation^28^. The detailed information of Biolog EcoPlate^TM^ is provided in Table S2 of SI. And the procedure of the Biolog EcoPlate^TM^ assay is described in Text S3 of SI.

### Illumina high-throughput sequencing

The microbial communities in the UASB reactors were identified by Illumina MiSeq high-throughput sequencing. The detailed information on DNA extraction, PCR amplification, the multiplexed DNA libraries construction and sequences fragments analysis were described in Text S4 of SI following Pan *et al*.^26^, and the Illumina MiSeq high-throughput sequencing was conducted by Personal Biotechnology Co., Ltd. (Shanghai, China).

### Functional microbial strain analysis

Functional strain analysis of the bacteria with X-3B degrading ability was carried out from the MLSS collected from G1-G5. And the method of bacterial isolation by Hamaki^59^ was used with minor modification. The strains were subjected to decolorization ability assessment by measuring the decolorization and growth curves, and the morphology of strains was analysed on a ZEISS ULTRA 55 FE-SEM (ZEISS, Oberkochen, Germany). In the end, the phylogenetic characterization of the strains was performed by 16S rRNA gene sequencing. The detailed information is shown in Text S5 of SI.

The statistical and bioinformatics analysis were also performed according to procedures described in our prior work^26^. All the assays were conducted in triplicate.

## Additional Information

**How to cite this article:** Pan, F. *et al*. Nanoscale zero-valent iron/persulfate enhanced upflow anaerobic sludge blanket reactor for dye removal: Insight into microbial metabolism and microbial community. *Sci. Rep.* **7**, 44626; doi: 10.1038/srep44626 (2017).

**Publisher's note:** Springer Nature remains neutral with regard to jurisdictional claims in published maps and institutional affiliations.

## Supplementary Material

Supplementary Information

## Figures and Tables

**Table 1:** COD removal efficiency, decolorization ratio, influent COD and effluent COD in stable operation stage of UASB.

	COD removal efficiency (%)	Decolorization ratio (%)	COD in influent (g/L•d)	COD in effluent (g/L•d)
G1 (Control)	87.30 ± 2.62	63.82 ± 1.9	4.0	0.51
G2 (0.1 g/L)	89.87 ± 2.70	93.89 ± 2.8	4.0	0.41
G3 (0.2 g/L)	90.05 ± 2.71	96.01 ± 2.9	4.0	0.40
G4 (0.5 g/L)	93.53 ± 2.79	98.38 ± 3.0	4.0	0.26
G5 (1.0 g/L)	88.57 ± 2.66	90.64 ± 2.7	4.0	0.46

**Table 2:** Alpha Richness and diversity estimators of the bacteria phylotypes in UASB.

Group	Chao1^a^	Ace^a^	Simpson^b^	Shannon^b^	Coverage^c^
Control	2737.97546	2837.173948	0.051957	4.44114	0.987199
G3 NZVI (0.2 g/L)	5179.35714	6262.711121	0.076037	4.628195	0.985415
G5 NZVI (1.0 g/L)	3418.33745	3739.0899	0.066444	4.026756	0.986813

^a^Chao1/Ace richness estimator: the total number of OTUs estimated by infinite sampling. A higher number indicates higher richness.

^b^Simpson/Shannon diversity index: an index to characterize species diversity. A higher value represents more diversity.

^c^Good’s coverage: estimated probability that the next read will belong to an OTU that has already been found.
