# Supplementary material for: Nanoscale zero-valent iron/persulfate enhanced upflow anaerobic sludge blanket reactor for dye removal: Insight into microbial metabolism and microbial community
Source: Sci Rep. 2017 Mar 16;7:44626. doi: 10.1038/srep44626 (PMC5353662; doi:10.1038/srep44626)
Supplement: Supplementary Information [file srep44626-s1.pdf]

Supplementary Information for

Nanoscale zero-valent iron/persulfate enhanced upflow anaerobic sludge blanket reactor for dye removal: Insight into microbial metabolism and microbial community

Fei Pan[1,2,*], Xiaohan Zhong[1], Dongsheng Xia[1], Xianze Yin[3], Fan Li[4], Dongye Zhao[4], Haodong Ji[4], Wen Liu [5,*]

[1]School of Environmental Engineering, Wuhan Textile University, Wuhan, 430073, P.R.China

[2]Engineering Research Centre for Clean Production of Textile Dyeing and Printing, Ministry of Education, Wuhan, 430073, P.R.China

[3]School of Materials Science and Engineering, Wuhan Textile University, Wuhan, 430073, P.R.China

[4]Environmental Engineering Program, Department of Civil Engineering, Auburn University, Auburn, AL 36849, USA

[5]School of Civil and Environmental Engineering, Georgia Institute of Technology, Atlanta, GA 30332, USA

***Corresponding authors**: Address: 1 Fangzhi Road, School of Environmental Engineering, Wuhan Textile University, Wuhan, 430073, P.R.China. Tel. & Fax: +86-027-59367338. E-mail address: fpan@wtu.edu.cn (F. Pan). Or Daniel Lab 304, School of Civil and Environmental Engineering, Georgia Institute of Technology, Atlanta, GA 30332, USA. E-mail address: wen.liu@ce.gatech.edu (W. Liu).

Text S1. The parameter of the UASB reactor

The UASB reactor was composed of a plexiglass column (D) with an internal diameter of 70 mm and an overall height of 500 mm, and a three-phase separator was fitted on top of the reactor. The effective volume of UASB reactor was 1.8 L. The dyeing wastewater was injected into the UASB reactor from the bottom via a peristaltic pump (C) at a liquid upflow speed of 1.0 m/h, and the produced gas could escape from an upper tube separated by the three-phase separator at the top of UASB reactor.

Text S2. The detailed information of inoculated sludge and synthetic dyeing wastewater

Inoculated sludge was taken from anaerobic digesters of the sewage treatment plant based at Wuhan Textile University (Wuhan, China). The inoculated sludge was acclimated by mixing with reactive brilliant red X-3B dye at concentration of 20 mg/L. The sludge was then incubated for 36 days so that it was able to degrade the reactive brilliant red X-3B dye. The inoculated sludge exhibited sizes of 1.0~3.0 mm (Figure S8, Supporting Information) and pH of 6.5~7.2. The inoculated sludge included 4088±50 mg/L of mixed liquor suspended solids (MLSS) and 2660 ±50 mg/L of mixed liquor volatile suspended solids (MLVSS). The influent dyeing wastewater was synthesized with reactive brilliant red X-3B dye using the following supply solution: sucrose 90 g/L, $K_2HPO_4 \cdot 3H_2O$ 1.62 g/L, KH_2PO_4 0.97 g/L, NH_4HCO_3 14.1 g/L, and $NaHCO_3$ 33.3 g/L. The chemical oxygen demand (COD) level of the dyeing wastewater was measured to be ca. 100 g/L. Dyeing wastewater was diluted to get the desired COD concentration before experiments.

Text S3. The detailed information of procedure for Biolog EcoPlate^{TM} assay

For a test, 10 mL of mixed liquor was centrifuged at 10000 rpm for 20 min and the supernatant was discarded. A 1 mL of saline was added to the residue. After vibration on a shaker for 5 min, the slurry was then centrifuged at 10000 rpm for 20 min and the supernatant was discarded. Repeat twice to remove the carbon sources. A 1 mL of saline was added to the residue. After vibration on a shaker for 5 min, the slurry was centrifuged at 2000 rpm for 1 min. The supernatant was poured into a tube with 20 mL sterile saline solution and allowed the optical density of 590 nm (OD_{590}) at 0.13 $\pm$0.02. The microbial suspension was then inoculated into the Biology EcoPlate with 150 μL per well. The inoculated plate was incubated in dark at 28 ℃. The color development in each well was continuously measured every 24 h for 7 days in terms of OD_{590} with a SpectraMax® Plus384 Absorbance Microplate Reader (Molecular Devices, Sunnyvale, CA, USA).

Text S4. The detailed information of illumina high-throughput sequencing

The microbial DNA of liquor samples drawn from the UASB reactors was isolated using an E.Z.N.A. Soil DNA Kit (Omega Bio-Tek, Norcross, GA, USA). The DNA extracts were then stored at -20 °C for PCR amplification. The universal V4 regions of the 16S rRNA genes primers were set to forward 520F (5'-AYTGGGYDTAAAGNG-3') and reverse 802R (5'-TACNVGGGTATCT AATCC -3').[1] The extracted DNA fragments and adapters with tags were added between the forward and adapters primers. And the detailed information of PCR amplification was followed:

The PCR mixture contained 20 ng of DNA temple, 2.00 μL of 2.5 mM dNTPs, 1.00 μL of Forward primers, 1.00 μL of Reverse primers, 0.25 μL of 5U Q5 Polymerase, 5.0 μL of 5 × Q5 GC high Enhancer and 5.0 μL of 5 × Q5 Reaction Buffer. Sterile double-distilled H_2O was performed via PCR amplification at a total volume of 25 μL. The PCR amplification condition was: an initial 30 s denaturation at 98 °C followed by 25 cycles of denaturing at 98 °C for 30 s, annealing at 50 °C for 30 s, extension at 72 °C for 30 s, and a final extension at 72 °C for 5 min.

The electrophoresis results of all of the amplicons, which were detected by 0.8 % agarose gels with a loading level of 3 μL, were satisfactory with clear strap (Figure S9, Supporting Information). The amplicons of PCR were purification and quantification, and the multiplexed DNA libraries were homogenized to 10 nM and were mixed at equal volumes. And then it was sent to Personal Biotechnology Co., Ltd. (Shanghai, China) for conducting the Illumina MiSeq high-throughput sequencing. And then the

obtained raw fastq were demultiplexed and quality-filtered via Qiime (version 1.7.0, http://qiime.org/) and Mothur (version 1.31.2, HTTP: //www.mothur.org/) to obtain high-quality sequences for subsequent analysis. Operational Taxonomic Units (OTUs) were also clustered with 97 % similarity using Qiime software. The high-quality sequences were classified to operational taxonomic units (OTUs) using Qiime software. The taxonomic information of each OTU was obtained from the comparison of sequences database using the blast method in Qiime. The rarefaction curve was constructed by random sampling for all the sequences, and the rank abundance curve was drawn to reflect the species distribution pattern.

Text S5. The detailed information of functional microbial strain analysis

Bacteria isolation was carried out from the MLSS collected from G1-G5. And the method of bacterial isolation was portrayed by Hamaki[2] with minor modification. The 150 g of dry MLSS was incubated in 300 mL of 50 mM NaOH overnight at room temperature. Then the mixture was centrifuged at 18000 ×g for 60 min and the supernatant was filtered with the membrane (0.2 μm) for a sludge-extract. The pH of the sludge-extract was controlled to 6.5. At last each 100 mL sludge-extract and 1 g agar were mixed and sterilized at 121 °C for 15 min. The sludge samples (5 g) which separately collected from G1-G5 were mixed with 50 mL of sterile distilled water (SDW) to form suspensions. Then serial dilutions (from 10^{-1} to 10^{-7}) were carried out and 50 μL of each dilution was spread into petri dish which containing sludge-extract and agar. The petri dishes which inoculated were incubated by inversion at 30 °C for 96h. Single colonies were haphazardly selected from the petri dishes, which characterized the richest phenotypes (e.g. brightness, margin, elevation, form, and color). And then the screen colonies were isolated by streak plate method and all of petri dishes which inoculated were incubated by inversion at 30 °C for 24h. Finally, pure single colonies were obtained and stored in glycerol at -70 °C.

Moreover, the microorganism species identification of isolated strains was carried out by 16S rRNA sequencing and phylogenetic analysis. The detailed information was followed:

The DNA of isolated strains was isolated using an Axygen® AxyPrep™ Bacterial

Genomic DNA Miniprep Kit (Product #AP-MN-BT-GDNA-50) (Corning, NY, USA). The microbial DNA extracts were then stored at -20 °C for PCR amplification. The universal V4 regions of the 16S rRNA genes primers were set to 27F (5'-AGAGTTTGATCCTGGCTCAG-3') and 1492R (5'- CTACGG CTACCTTGTTACGA -3'). The PCR mixture contained 20 ng of DNA temple, 1.00 μL of 10 mM dNTPs, 1.50 μL of Forward primers, 1.50 μL of Reverse primers, 1.0 μL of 5U Taq Polymerase and 5.0 μL of 10 × Buffer (2.5mM Mg^{2+}). Sterile double-distilled H_2O was performed via PCR amplification at a total volume of 50 μL. The PCR amplification condition was: an initial 5 min denaturation at 95 °C followed by 35 cycles of denaturing at 95 °C for 30 s, annealing at 58 °C for 30 s, extension at 72 °C for 90 s, and a final extension at 72 °C for 7 min. Then, the sequences obtained from isolated strains were compared with the data in GenBank database of National Centre for Biotechnology Information (NCBI) by employing BLAST tools (http://blast.ncbi.nlm.nih.gov/ Blast.cgi) and the phylogenetic trees were mapped.

In the end, the strains KX421197 (FP-A1), KX421198 (FP-B1) and KX421199 (FP-C1) were incubated at 30 °C without shaking. Culture media which used for the three strains growth were as follows. Luria-Bertani medium consisted of 5 g/L yeast extract, 5 g/L NaCl and 10 g/L peptone[2]. Moreover, the biological degradation of reactive brilliant red X-3B by three strains was also investigated; 50 mg/L reactive brilliant red X-3B was added into sludge-extract and the decolourization ratio was monitored by measuring the optical density (OD) at 540 nm[3]. The growth of three

strains was monitored by measuring the optical density (OD) at 600 nm[4] and the result was shown in Figure 5 and Figure S5,S6 and S7.

Reference:

1. Pan, F.*et al*. Effects of octahedral molecular sieve on treatment performance, microbial metabolism, and microbial community in expanded granular sludge bed reactor. *Water Res.* **87**, 127-136(2015).
2. Hamaki, T. *et al*. Isolation of novel bacteria and actinomycetes using soil-extract agar medium. *J Biosci Bioeng* **99**, (5), 485-492(2005).
3. Pan, F.; Luo, Y.; Zhang, L.R.; Fu, J., Degradation of Reactive Brilliant Red X-3B by zero-valent iron/activated carbon system in the presence of microwave irradiation. *Water Sci Technol* **64**, (12), 2345-2351(2011).
4. Pal, S.; Tak, Y. K.; Song, J. M., Does the Antibacterial Activity of Silver Nanoparticles Depend on the Shape of the Nanoparticle? A Study of the Gram-Negative Bacterium Escherichia coli. *Appl Environ Microbiol* **73**, (6), 1712-1720(2007).

Table S1. The experimental schedule for influent COD and X-3B loading of UASB

Phase	Time (d)	Influent COD (g/m^3•d)	X-3B loading (g/m^3•d)
		G1 – G5	G1 – G5
**1:Start-up**	0-62	4000 ± 142	2.5-25.6
**2:X-3B loading increase**	63-112	7000 ± 268	30.1-62.1
**3:Recovery**	113-131	3500 ± 129	18.6-35.7
**4:Stable operation**	132-208	4000 ± 138	23.7-27.8

Table S2. Individual carbon sources and their classification in Biolog EcoPlate^{TM}

Carbon source	Substrate	Chemical formula	Serial No.
Amines/amides (n= 2)	G4: Phenyethylamine	$C_8H_{11}N$	28
	H4: Putrescine	$C_4H_{12}N_2$	31
Amino acids (n= 6)	A4: L-Arginine	$C_6H_{14}N_4O_2$	4
	B4: L-Asparagine	$C_4H_8N_2O_8$	8
	C4: L-Phenylalanine	$C_9H_{11}NO_2$	12
	D4: L-Serine	$C_3H_7NO_3$	16
	E4: L-Threonine	$C_4H_9NO_3$	20
	F4: Glycyl-L-glutamic acid	$C_7H_{12}N_2O_5$	24
Carbohydrates (n= 7)	A2: β-Methyl-D-glucoside	$C_7H_{14}O_6$	2
	B2: D-Xylose	$C_5H_{10}O_5$	6
	C2: i-Erythritol	$C_4H_{10}O_4$	10
	D2: D-Mannitol	$C_6H_{14}O_6$	14
	E2: N-Acetyl-D-glucosamine	$C_8H_{15}NO_6$	18
	G1: D-Cellobiose	$C_{12}H_{22}O_{11}$	21
	H1: α-D-Lactose	$C_{12}H_{22}O_{11}$	25
Carboxylic acids (n= 9)	A3: D-Galactonic acid γ-Lactone	$C_6H_{10}O_6$	3
	B3: D-Galacturonic acid	$C_6H_{10}O_7$	7
	C3: 2-Hydroxy benzoic acid	$C_7H_6O_3$	11
	D3: 4-Hydroxy benzoic acid	$C_7H_6O_3$	15
	E3: γ-Hydroxy butyric acid	$C_4H_8O_3$	19
	F2: D-Glucosaminic acid	$C_6H_{13}NO_6$	22
	F3: Itaconic acid	$C_5H_6O_4$	23
	G3: α-Keto butyric acid	$C_4H_6O_3$	27
	H3: D-Malic acid	$C_4H_6O_5$	30
Miscellaneous (n= 3)	B1: Pyruvic acid methyl ester	$C_4H_6O_3$	1
	G2: Glucose -l-phosphate	$C_6H_{13}O_9P$	26
	H2: D,L-α-Glycerol-phosphate	$C_3H_9O_6P$	29
Polymers (n = 4)	E1: α-Cyclodextrin	$C_{36}H_{60}O_{30}$	13
	F1: Glycogen	$(C_6H_{10}O_5)_n$	17
	C1: Tween 40	-	5
	D1: Tween 80	-	9

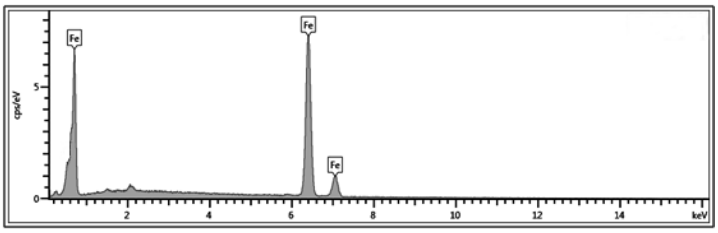

A

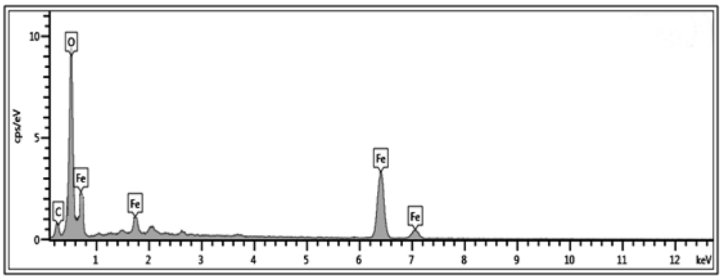

B

Figure S1. The EDS spectra of fresh NZVI **(A)** and used NZVI in UASB reactors for 30 days **(B)**

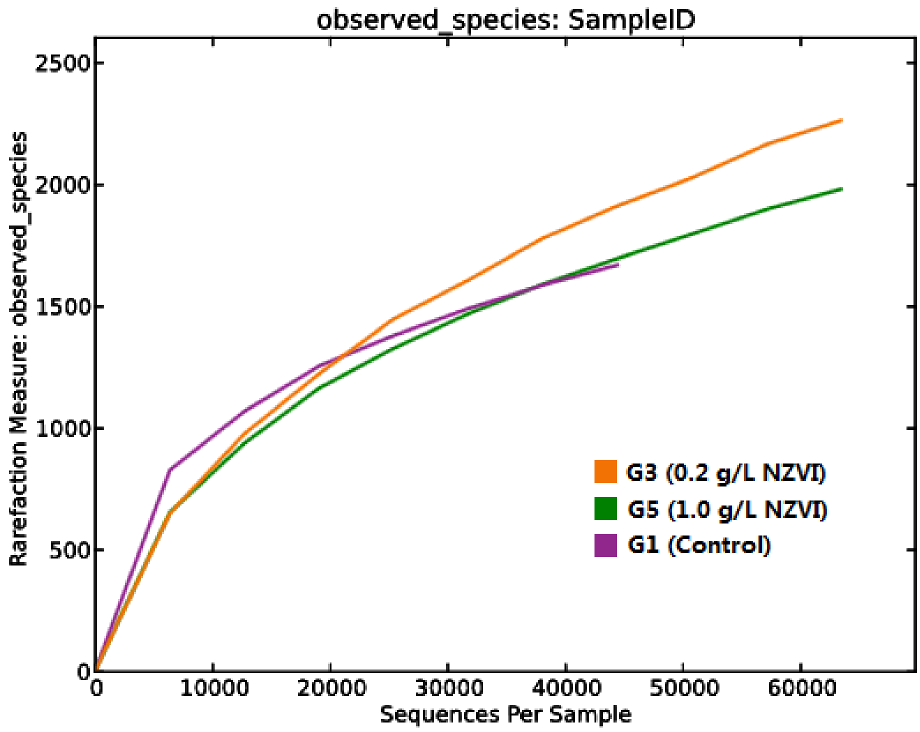

Figure S2. The rarefaction curves of G1(Control), G3(0.2 g/L NZVI) and G5(1.0 g/L NZVI) samples in UASB reactors.

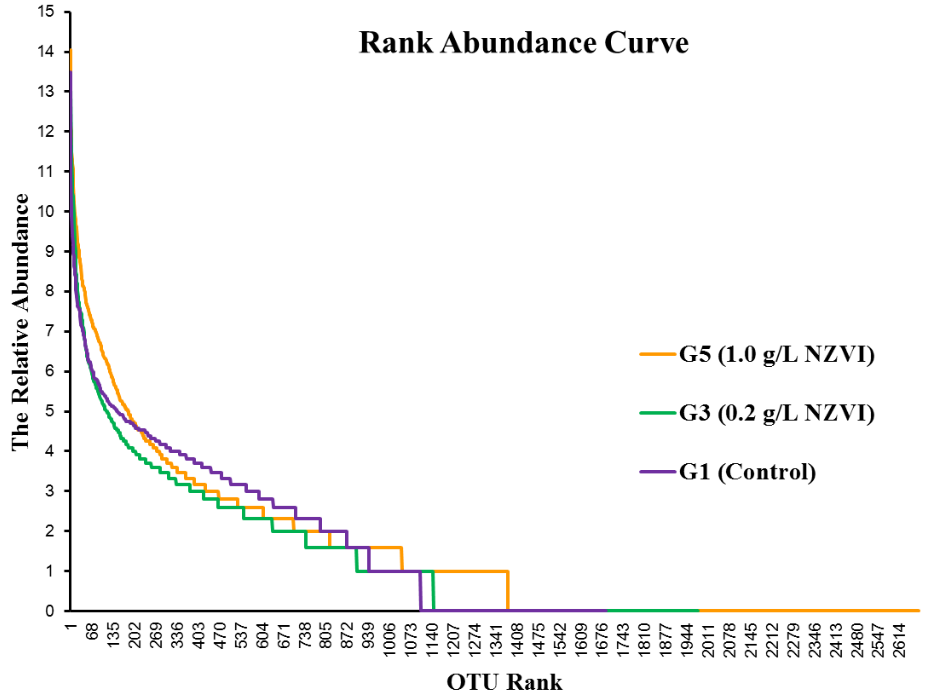

Figure S3. The rank abundance curves of G1(Control), G3(0.2 g/L NZVI) and G5(1.0 g/L NZVI) samples in UASB reactors.

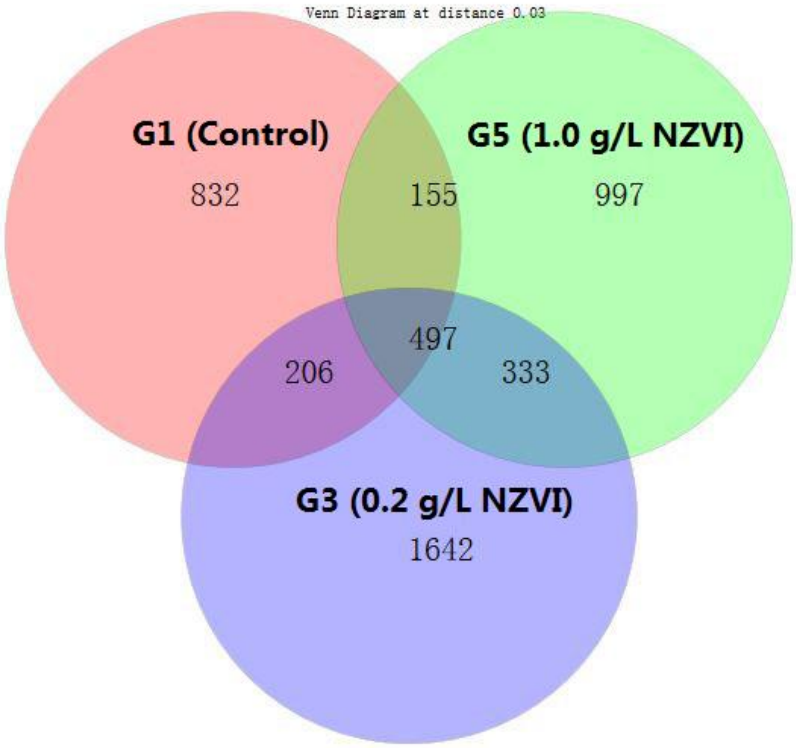

Figure S4. The Venn diagram of OTUs in G1(Control), G3(0.2 g/L NZVI) and G5(1.0 g/L NZVI) samples.

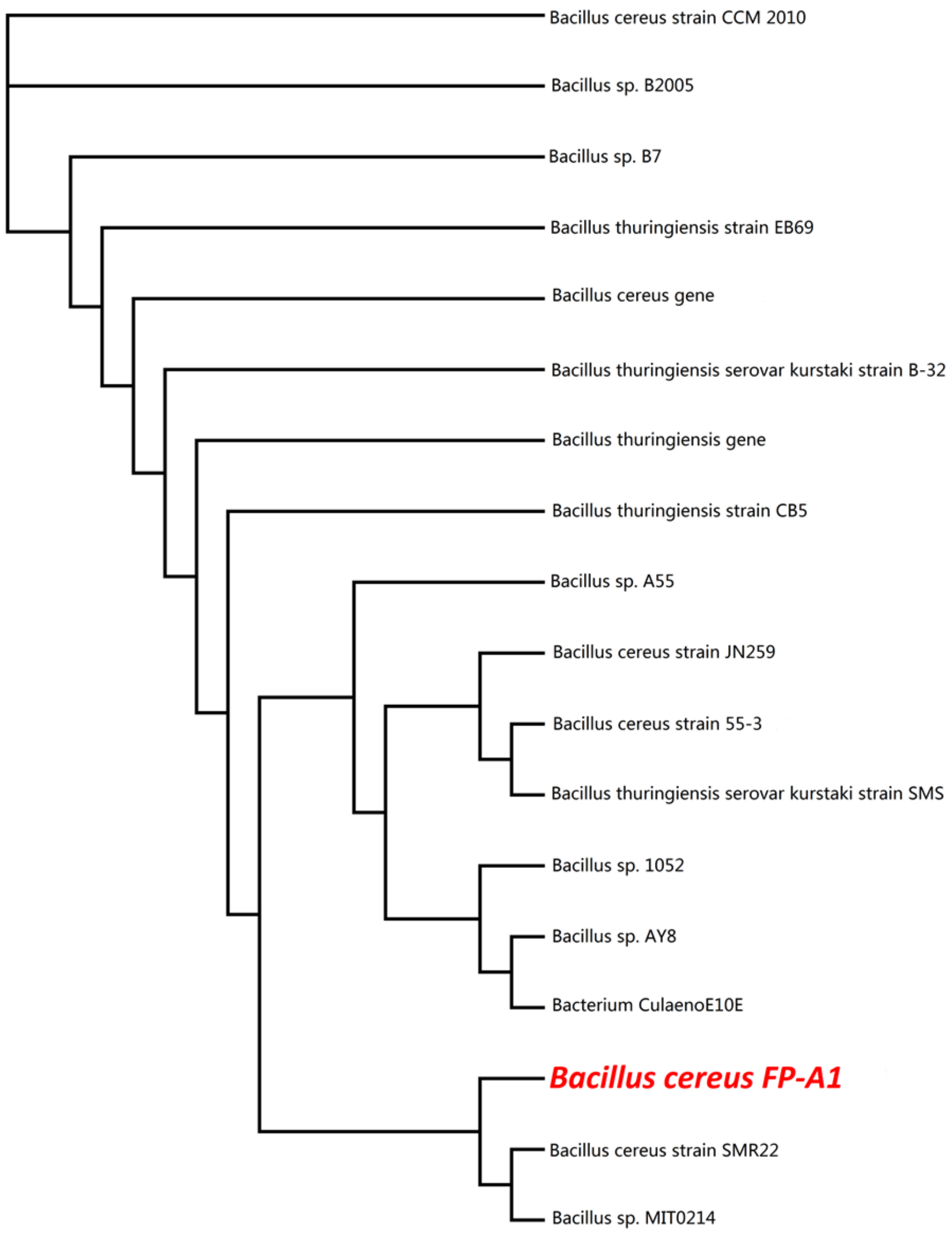

Figure S5. The phylogenetic tree of KX421197 (FP-A1).

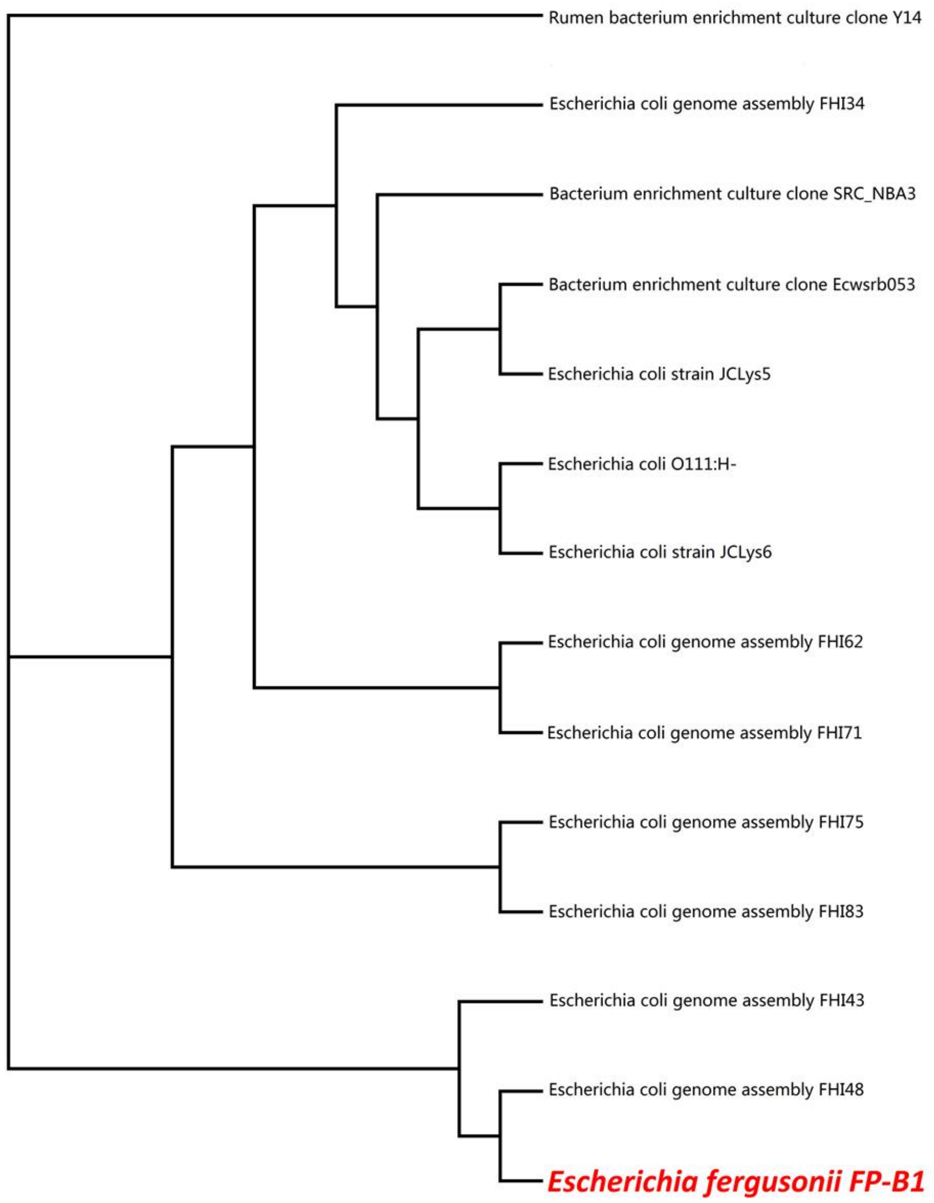

Figure S6. The phylogenetic tree of KX421198 (FP-B1).

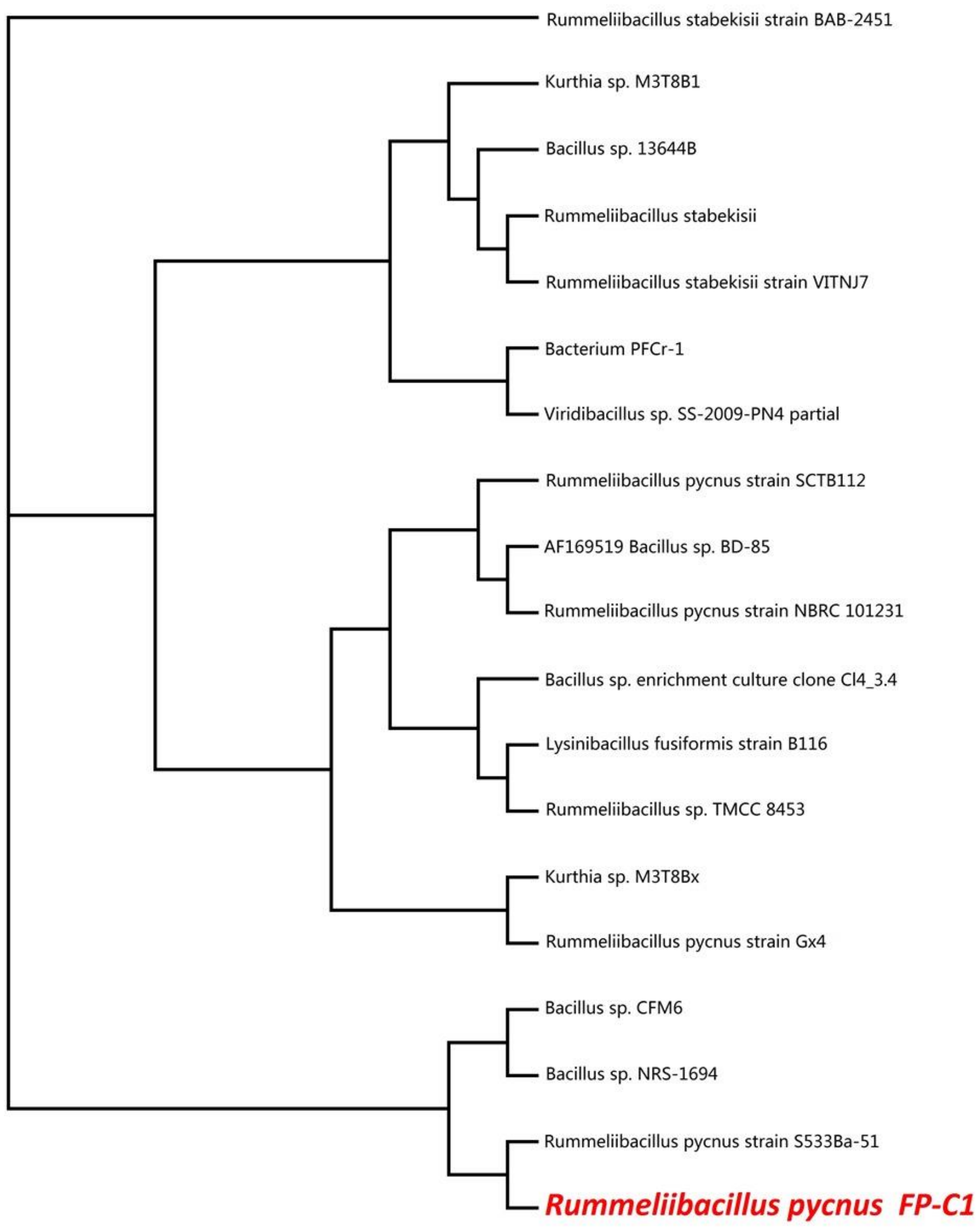

Figure S7. The phylogenetic tree KX421199 (FP-C1).

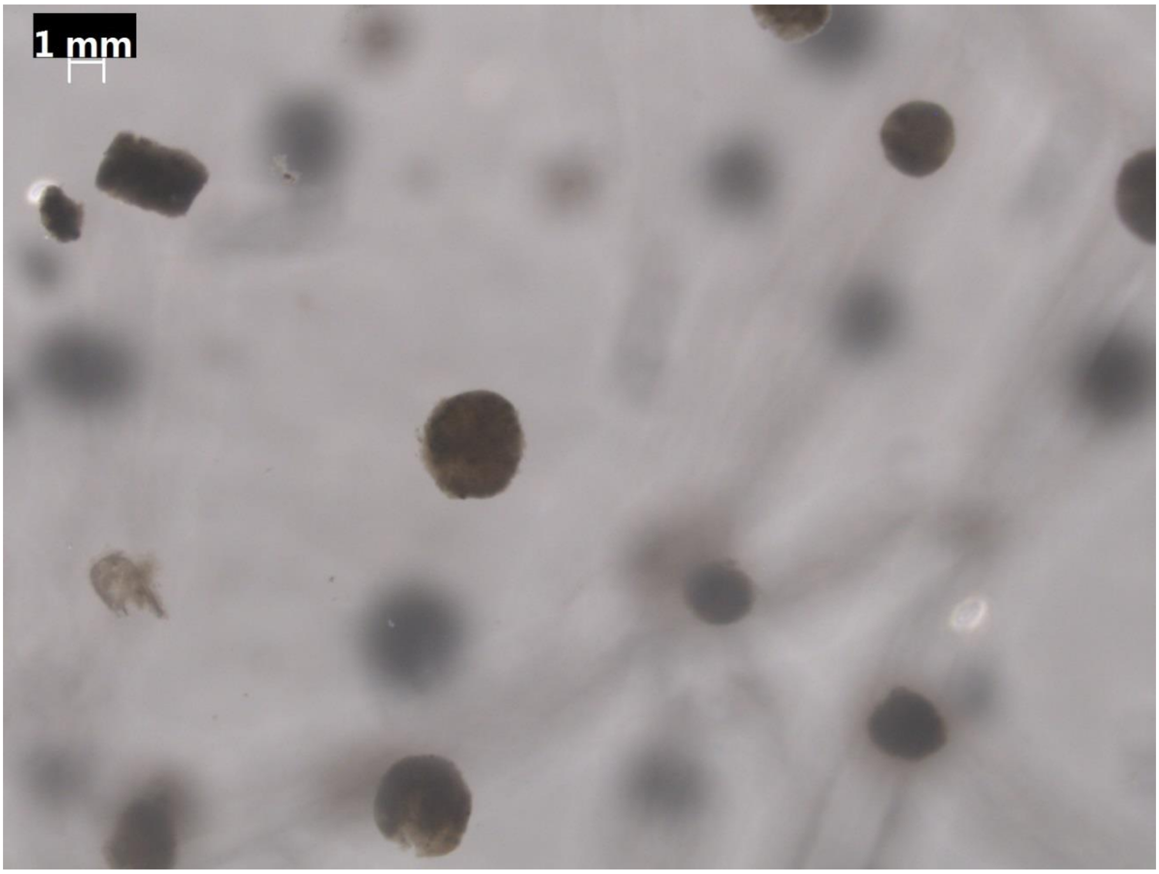

Figure S8. Morphology of the granular sludge in the steady stage of UASB reactors.

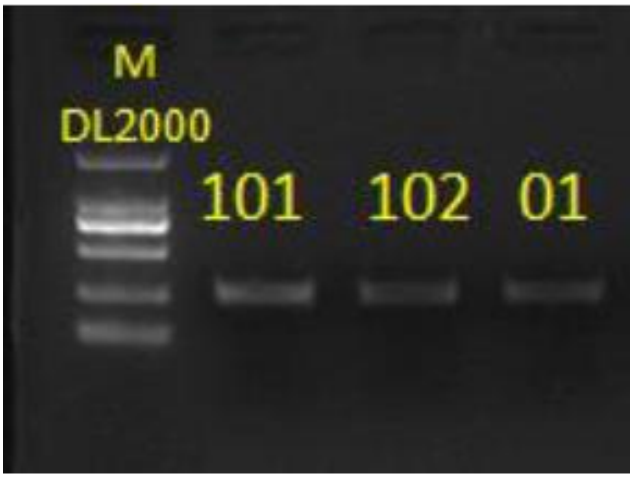

Figure S9. The electrophoresis results of all the amplicons. 01: G1 (Control), 101: G3 (0.2 g/L NZVI) and 102: G5 (1.0 g/L NZVI).
